# Supplementary material for: FGF2 alters macrophage polarization, tumour immunity and growth and can be targeted during radiotherapy
Source: Nat Commun. 2020 Aug 13;11:4064. doi: 10.1038/s41467-020-17914-x (PMC7426415; doi:10.1038/s41467-020-17914-x)

Supplementary Information for:

FGF2 Alters Macrophage Polarization, Tumour Immunity and Growth and can be Targeted during Radiotherapy

Im et al.

Supplementary Figure 1

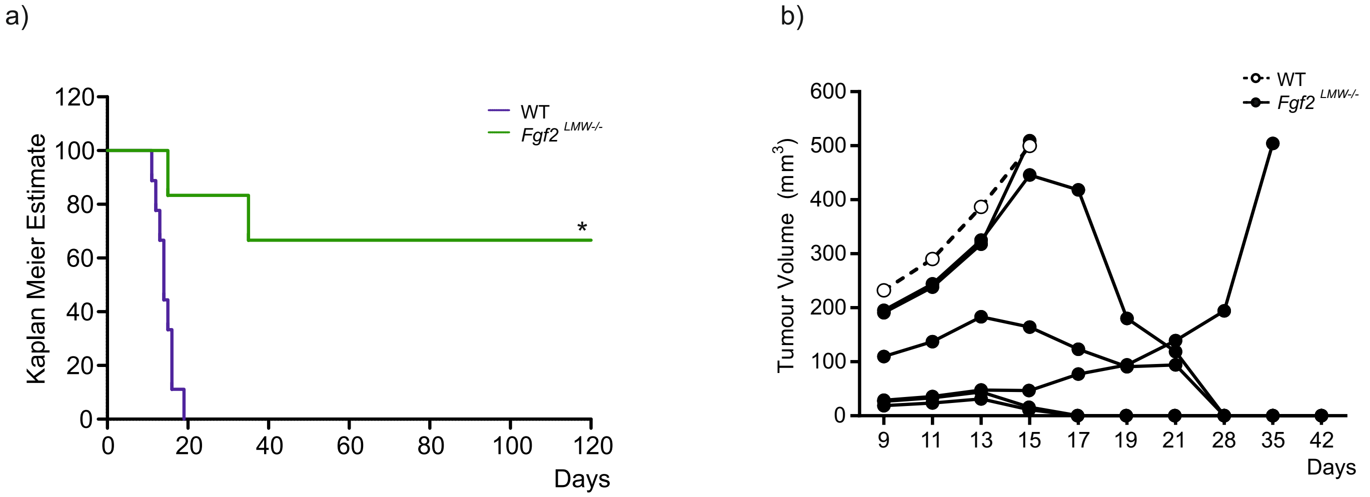

Supplementary Figure 1: Liver metastasis model

a) Kaplan Meier Estimate and (b) tumour growth curves following LLC inoculation in WT and *Fgf2*$^{LMW-/-}$ mice N=6 in each group. * represents statistical significance ($p \leq 0.05$) using Kaplan-Meier estimates. Error bars indicate S.D.

Supplementary Figure 2

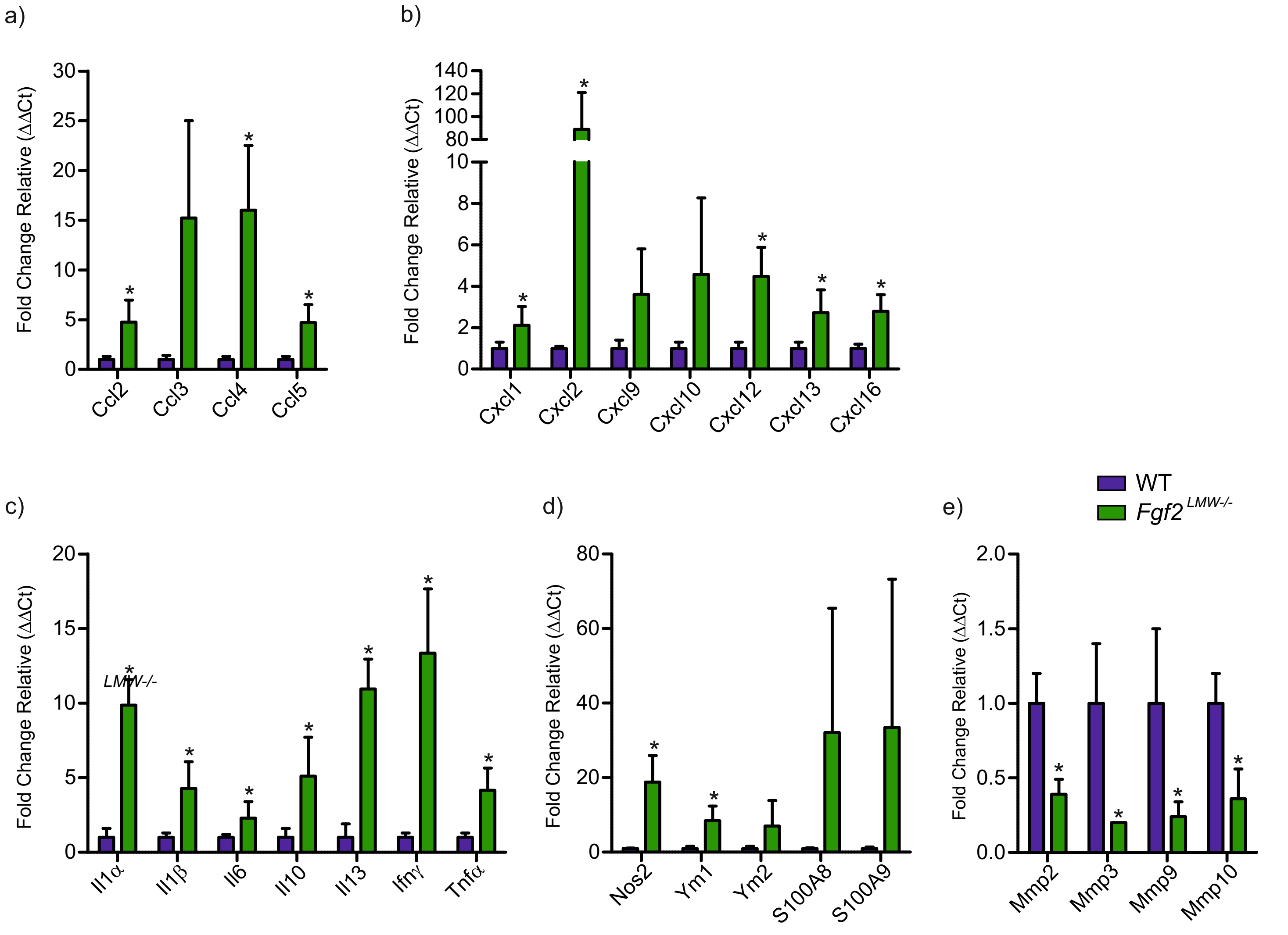

Supplementary Figure 2: Chemokine and cytokine expression tumour of Fgf2 $^{LMW-/-}$ *mice*

RNA expression of (a) CCLs, (b) CXCLs, (c) cytokines, (d) myeloid genes and (e) MMPs in subcutaneous tumours of *Fgf2* $^{LMW-/-}$ mice. * represents statistical significance ($p \leq 0.05$) using Kruskal-Wallis one-way ANOVA. Error bars indicate S.D. N=6 in WT and n=9 in of *Fgf2* $^{LMW-/-}$ mice.

Supplementary Figure 3

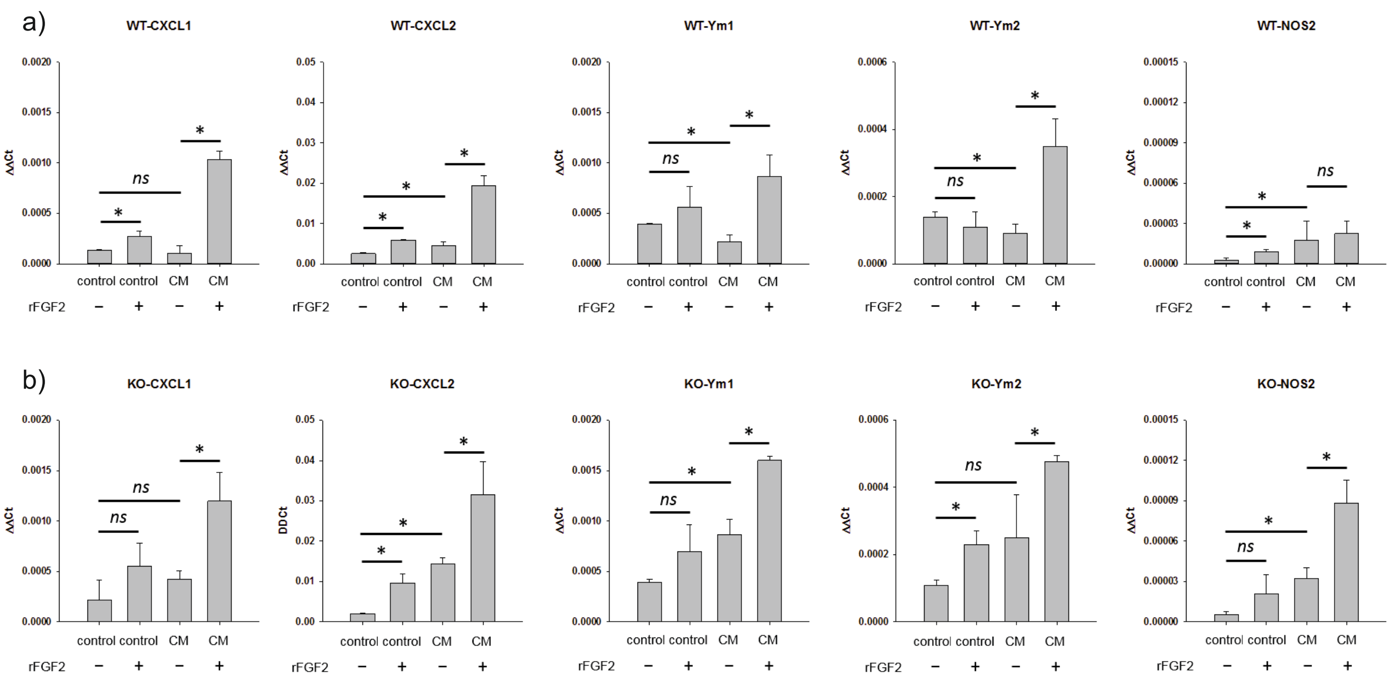

Supplementary Figure 3: Gene expression in BMDM from WT and Fgf2$^{LMW-/-}$ mice

BMDM from: (a) WT mice, and (b) *Fgf2*$^{LMW-/-}$ mice were subjected to analysis by qPCR for CXCL1, CXCL2, Ym1, Ym2 and NOS2 following addition of MC38 CM and rFGF2 (100 ng/ml). Control medium was changed to 50 % MC38 conditioned medium. After incubation for 5 days rFGF2 (Cat No. 710304, Biolegend) was added in serum-free medium for 1 h and then incubated for 24 h under serum-starvation. DNA was synthesized with RNA collected from BMDM and applied to qPCR analysis (12.5 ng/10.5 µl). * represents statistical significance ($p \leq 0.05$) using the Mann-Whitney test. Error bars indicate S.D.

Supplementary Figure 4

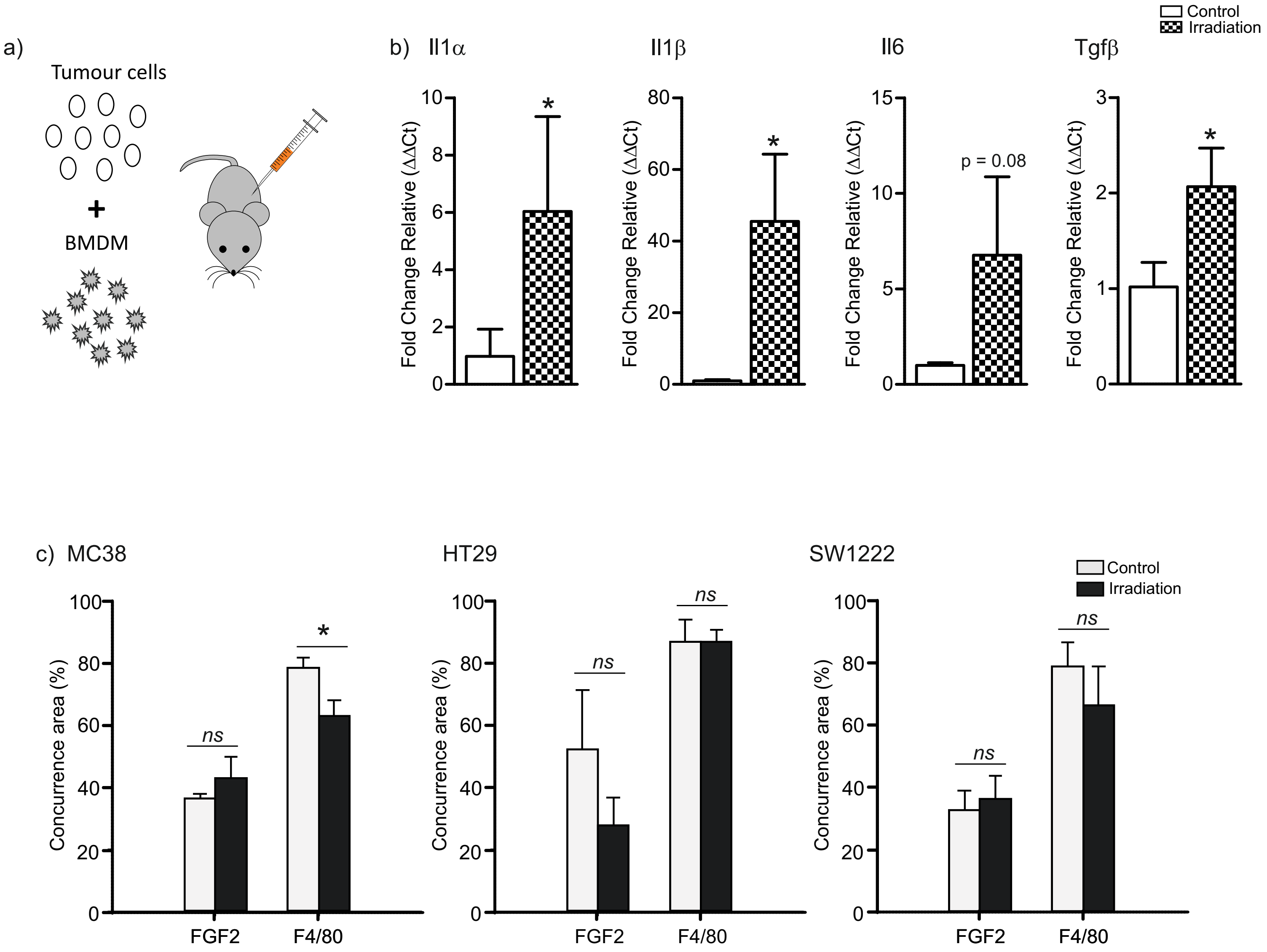

Supplementary Figure 4: BMDM activation following co-culture with tumour cells and quantification of concurrence between FGF2 and F4/80

(A) Depiction of subcutaneous co-injection model, and (B) qPCR analysis of (i) Il1 , (ii) Il1 , (iii) IL6 and (iv) Tgf following co-culturing of BMDM with tumour cells. *represents statistical significance ($p \leq 0.05$) using Kruskal-Wallis one-way ANOVA. Error bars indicate S.D. (C) Concurrence quantification of FGF2 and F4/80 in Fig. 6B. 'FGF2' in x-axis indicates the fraction of FGF2 co-localising with F4/80 out of the total FGF2 stained area. 'F4/80' refers to the fraction of F4/80 co-localising with FGF2. Quantification was examined with the images calculator of ImageJ. *represents statistical significance ($p \leq 0.05$) using the Mann-Whitney test. Error bars indicate S.D.

Supplementary Figure 5

a) MC38 with *Fgf2* $^{LMW-/-}$ mice

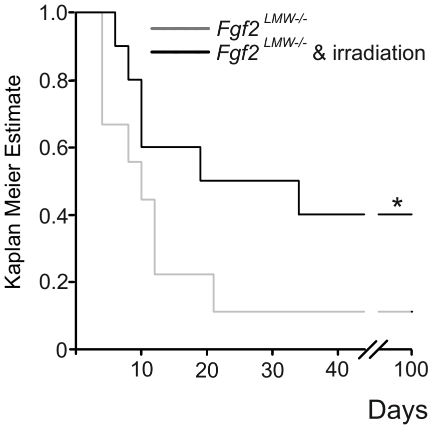

b) MC38 with wild type mice

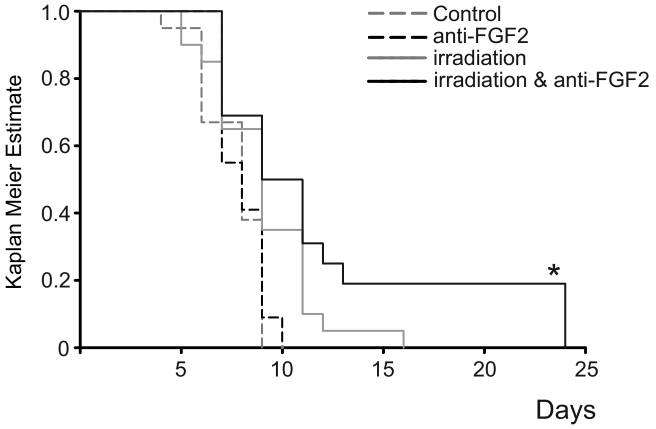

c) SW122

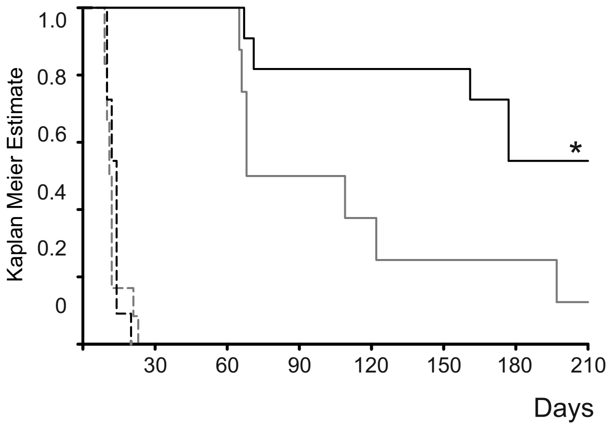

d) HT29

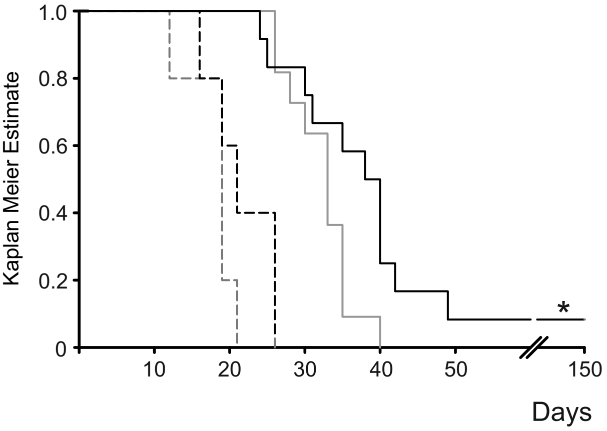

e)

Mice for tumour growth experiments (Figure 7)				
EXP	WT mice			FGF LMW$^{-/-}$ mice
	MC38	SW1222	HT29	MC38
Control	21	16	14	7
FGF2 mAb	22	15	15	
9x2 Gy	32	28	22	10
9x2 Gy + FGF2 mAb	38	10	22	

Mice for vascular staining experiments (Supp. Figure 8)				
EXP	MC38		SW1222	
	mice no	vessel no	mice no	vessel no
Control	5	7580	6	3102
FGF2 mAb	5	5054	5	2973
9x2 Gy	6	6309	5	1882
9x2 Gy+FGF2 mAb	6	2481	5	1203

f)

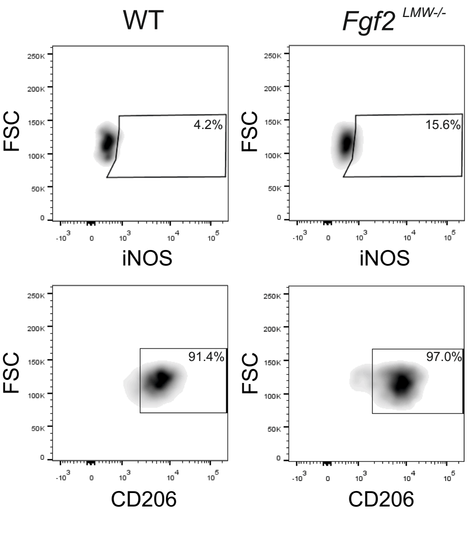

Supplementary Figure 5: Survival estimates following irradiation, anti-FGF2 combination therapy

Kaplan Meier Estimates of MC38 in (a) *Fgf2* $^{LMW-/-}$ mice and (b) WT mice, and (c) SW1222, (d) HT29 in Nu/Nu mice following anti-FGF2, irradiation or combination therapy. Irradiation to tumours was 9 times 2 Gy. These data are from experiments also shown in Figure 7. * represents statistical significance ($p \leq 0.05$) using Kaplan-Meier estimates. (e) Mice numbers analysed for Fig. 7 and Supplementary Fig. 8. (f) shows representative flow cytometry gating used in generating Fig. 4A.

Supplementary Figure 6

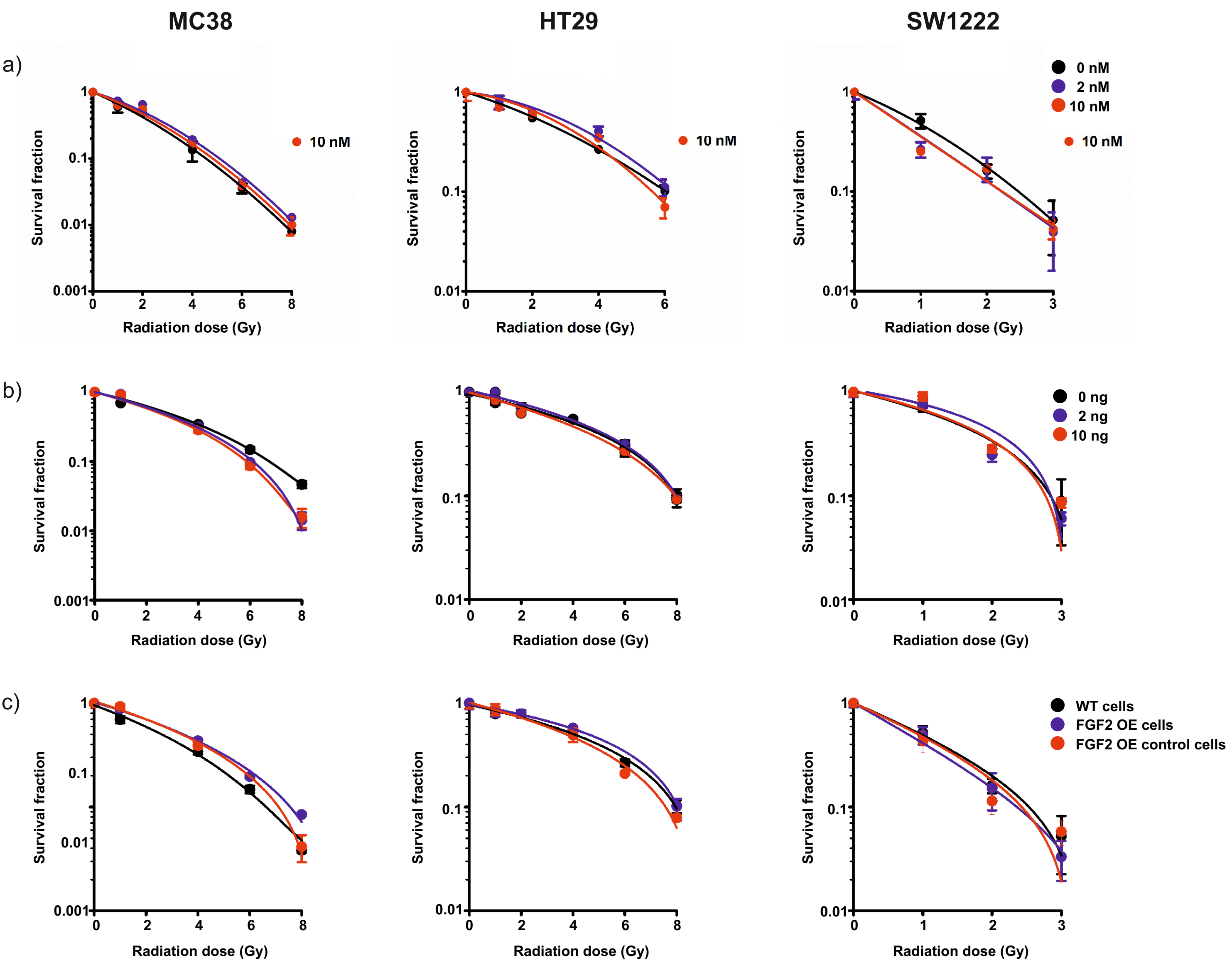

d)

	FGF2 mAb			BGJ398 (FGFR pan-inhibitor)		
	0	10 ng	40 ng	0	2 nM	10 nM
MC38	51.3±6.35	60.7±9.07	63.3±9.29	51.3±6.35	75.5±12.02	78.0±9.90
SW1222	22.6±10.29	26.3±4.73	18.3±0.58	22.6±10.29	32.0±7.07	29.5±17.68
HT29	54.0±4.05	41.3±0.58	50.7±4.73	54.0±4.05	66.1±7.07	62.5±16.26

Supplementary Figure 6: Radiation clonogenic survival assays with anti-FGF2 blocking antibody, FGFR inhibition and FGF2 overexpression

Survival fraction from radiation survival clonogenic assays in combination with (a) BGJ398 (pan FGFR inhibitor, Cat No. NVP-BGJ398, Selleckchem), (b) recombinant FGF2 and (c) cells transfected with a lentivirus expression vector for FGF2^{LMW}. (d) shows the plating efficiency of each of the cancer cells after these treatments at 0 Gy. Error bars indicate S.D.

Supplementary Figure 7

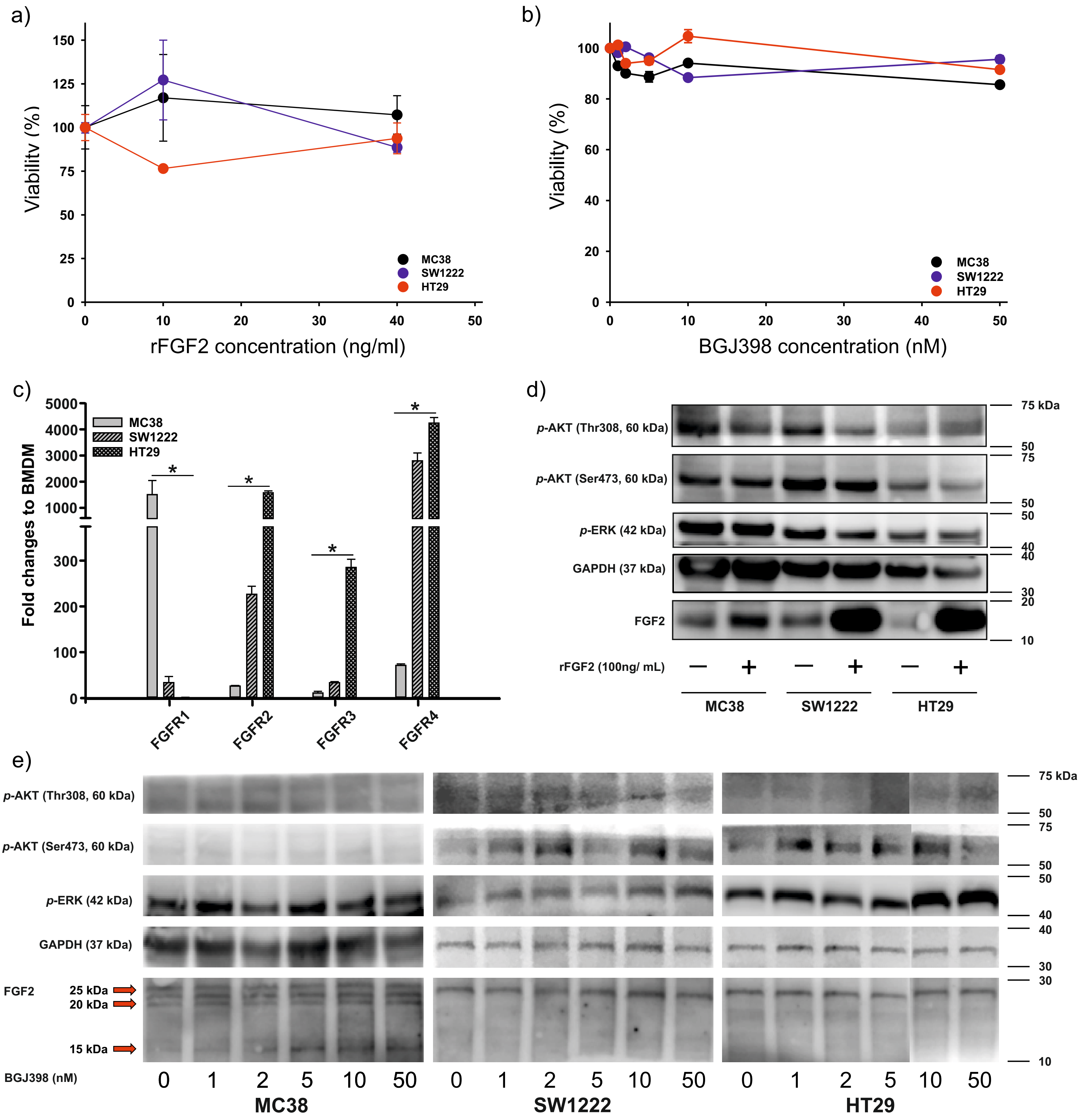

Supplementary Figure 7: Effect of FGF2 on signalling pathways in tumour cells

Viability of tumour cells at 48 h after addition of recombinant (a) FGF2 and (b) BGJ398, an panFGFR inhibitor. At 24 h after tumour cells (10 /well) were seeded in 6-well plates, rFGF2 or BGJ398 was added and incubated for 48 h. (c) qPCR analysis of FGF receptor genes in MC38, SW1222 and HT29. (d) Western blots with lysates of tumour cells after rFGF2 addition and blotting with indicated antibodies. Medium was changed to serum free at 24 h after tumour cells seeded. After incubation for 24 h, rFGF2 (100ng/ml) was added and incubated for 1 h. (e) Western blot with lysates of tumour cells after BGJ398 addition and blotted with the indicated antibodies. At 24 h after tumour cells were seeded, BGJ398 was added and incubated for 24 h. Images of the original gels are available as source data. * represents statistical significance ($p \leq 0.05$) using the Mann-Whitney test. Error bars indicate S.E.

Supplementary Figure 8

a) Vascular staining with CD31

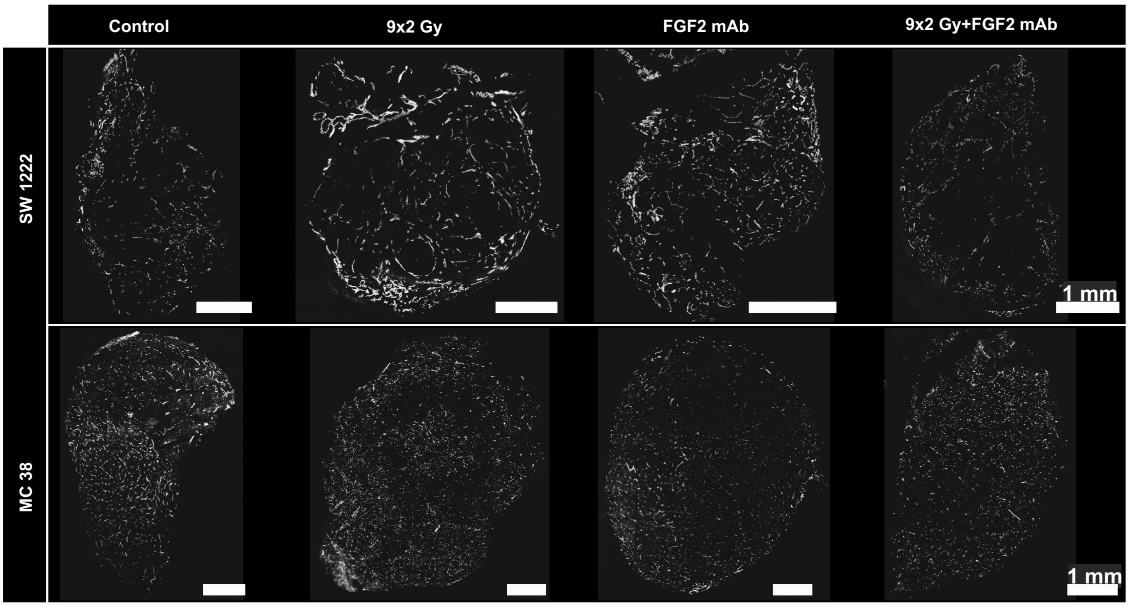

b) Vascular size distribution

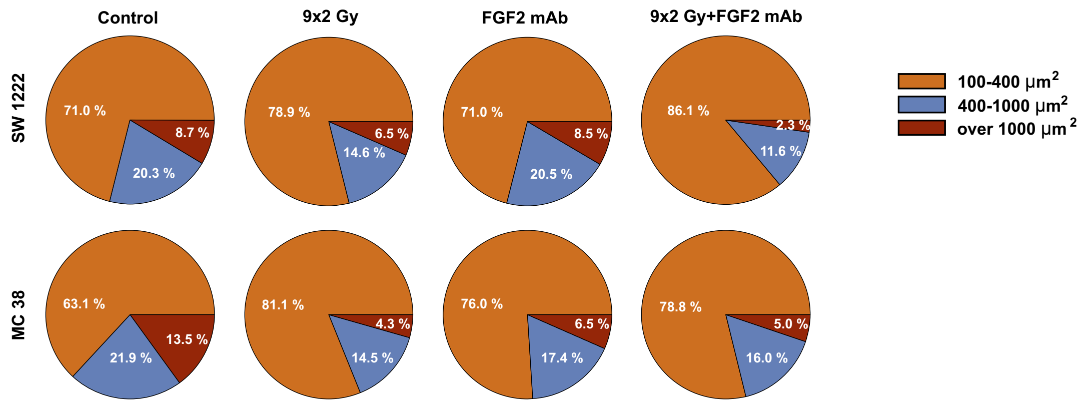

c) Average vascular size

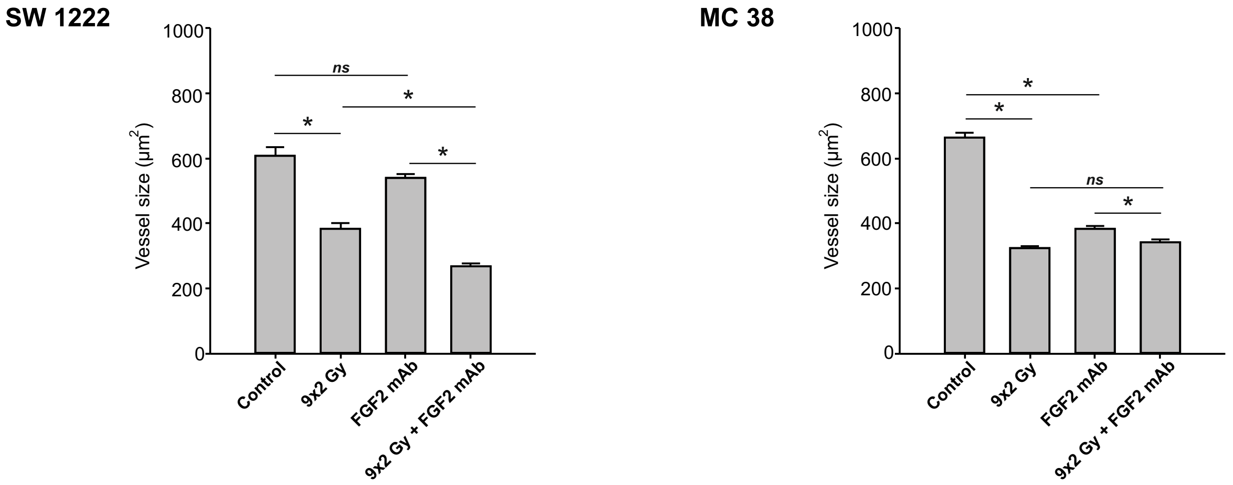

Supplementary Figure 8: Effect of irradiation and FGF2 blocking antibody on tumoral vessels

Sections across the entire tumours were stained with rat anti-CD31 antibody (Cat No. ab7388, Abcam, 1:100) and anti-rat Alexa fluor 546 (Cat No. A-11081, Invitrogen, 1:200). Whole tumour and vascular images were obtained using the tile scanning mode with a Zeiss 880 two-photon microscope (10x). The images were analysed with ImageJ; (a) tumoral vascular images, (b) analysis of vascular size distribution and (c) average vascular size. * represents statistical significance ($p \leq 0.05$) using the Mann-Whitney test. Error bars indicate S.E. Details of mice and vessel numbers are shown in Supplementary Fig. 5E table.

Supplementary Figure 9

a) 3D reconstruction of vessels in MC38 tumours

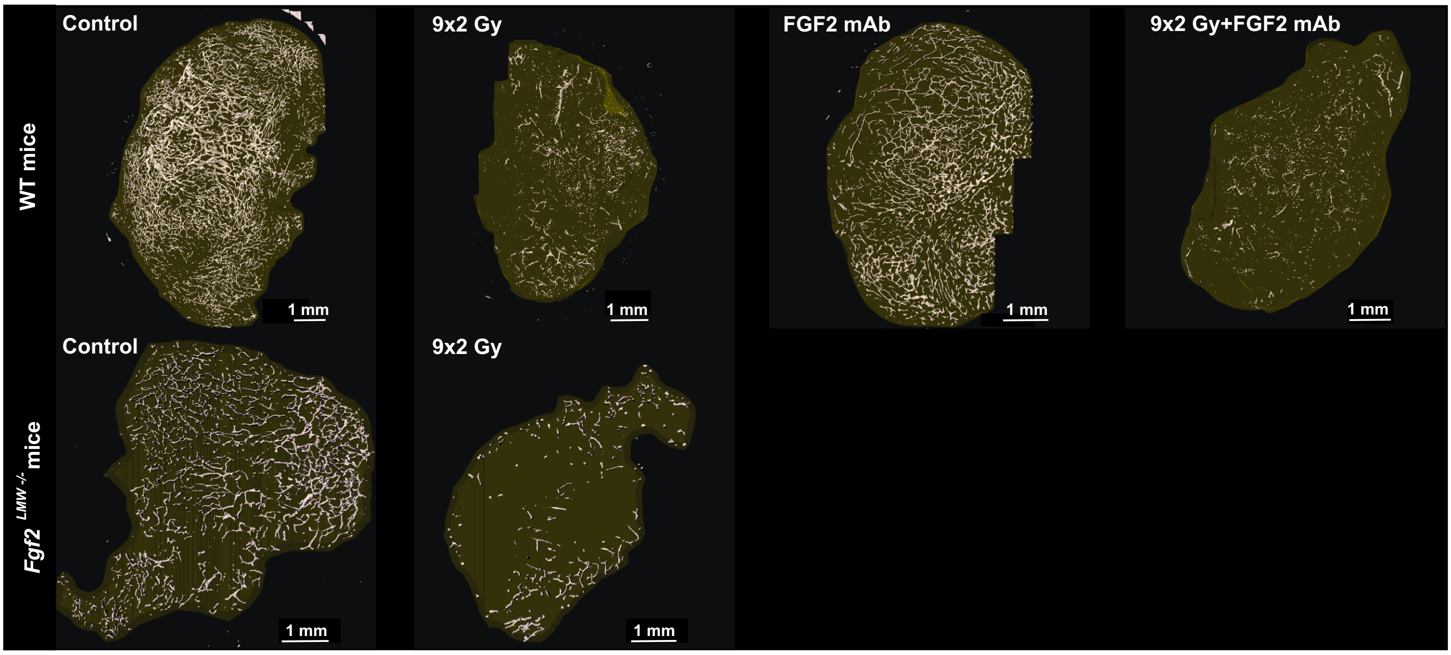

b) Vascular volume

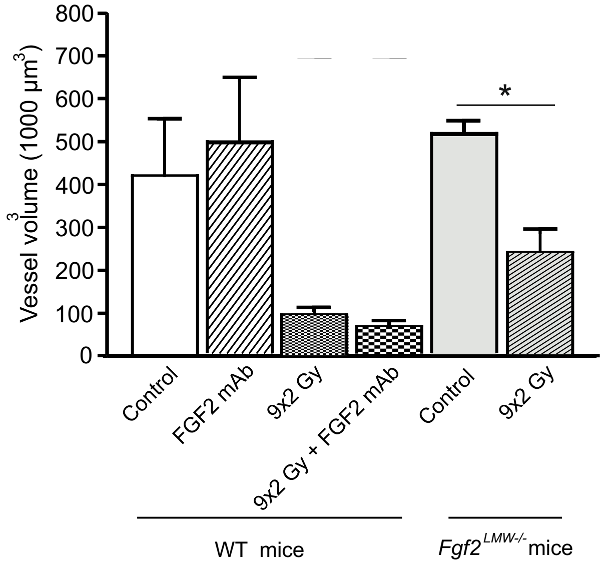

c) Experiment details

Mice for vascular staining experiments		
**EXP**	**WT mice**	***Fgf2*** $^{LMW-/-}$ **mice**
**Control**	**3**	**5**
**FGF2 mAb**	**3**	
**9x2 Gy**	**3**	**5**
**9x2 Gy + FGF2 mAb**	**4**	

Supplementary Figure 9: Effect of irradiation on tumour vessels in Fgf2 $^{LMW-/-}$ *mice*

Anti-CD31 antibody conjugated with PE (50 µl, Cat No. 102408, Biolegend) was I.V. injected at 5 min before tumour isolation. Vascular images were obtained with Zeiss 880 two-photon microscope, and the images were reconstructed in 3D and analysed with IMARIS (Oxford Instrument, UK): (a) 3D reconstructed tumoral vascular images, (b) analysis of vascular volume and (c) experimental details. * represents statistical significance ($p \leq 0.05$) using the Mann-Whitney test. Error bars indicate S.E.

Supplementary Table 1

Fluorochrome	Company	Catalogue Number
LIVE/DEAD™ Fixable Violet Dead Cell Stain Kit, for 405 nm excitation	Life Technologies	L34955
Alexa Fluor® 700 anti -mouse CD45 Antibody	BioLegend	103127
CD8a Monoclonal Antibody (53 -6.7), PE-Cyanine7	eBiosource	25-0081-82
CD11c Monoclonal Antibody (N418), PE	eBiosource	12-0114-82
Ly-6G (Gr-1) Monoclonal Antibody (RB6 -8C5), PerCP-Cyanine5.5	eBiosource	45-5931-80
CD3e Monoclonal Antibody (145 -2C11), APC	eBiosource	17-0031-82
Brilliant Violet 510™ anti -mouse/human CD11b Antibody	BioLegend	101245
Brilliant Violet 605™ anti -mouse CD4 Antibody	BioLegend	100547
APC/Cy7 anti -mouse F4/80 Antibody	BioLegend	123117
bFGF Polyclonal Antibody, FITC Conjugated	Bioss	bs -0217R-FITC
FGF R1 Antibody (M19B2) [Alexa Fluor® 488]	Novus	NB600-1287AF488
FGFR2 Polyclonal Antibody (conguated to alexa 488)	ThermoFisher	PA5-14651
iNOS Monoclonal Antibody (CXNFT), PE	eBiosource	12-5920-82
PerCP/Cy5.5 anti -mouse CD206 (MMR) Antibody	BioLegend	141716

Supplementary Table 2

Gene	Forward Primer	Reverse Primer
(RP) L32	GAGGTGCTGCTGATGTGC	GGCGTTGGGATTGGTGACT
Ccl2	TGTAGTTTTTGTCACCAAGCTCA	GTGCTTGAGGTGGTTGTGGA
Ccl3	GCCAGGTGTCATTTTCCTGAC	TCGATGTGGCTACTTGGCAG
Ccl4	CTGTGCAAACCTAACCCCGA	GGGTCAGAGCCCATTGGTG
Ccl5	CTTGCAGTCGTGTTTGTCACT	CCGAGTGGGAGTAGGGGATT
Cxcl1	CACCCAAACCGAAGTCATAGC	TTCTCCGTTACTTGGGGACACC
Cxcl2	AGTGAACTGCGCTGTCAATG	TTCAGGGTCAAGGCAAACTT
Cxcl9	CAGCTCTGCCATGAAGTCCG	AGGGTTCCTCGAACTCCACAC
CxCl10	TCATCCTGCTGGGTCTGAGT	CATCGTGGCAATGATCTCAACA
Cxcl12	CTTCAGATTGTTGCACGGCT	GCGAGTTACAAAGCGCCAGA
Cxcl13	CTCTCTCCAGGCCACGGTATT	TTTGGCACGAGGATTCACAC
Cxcl16	TGCAGGTTTGCAGCTCTGGTA	AAGGGTCAGCAGGTCAAAGC
IfnI	TGCCACGGCACAGTCATT	CCAGTTCCTCCAGATATCCAAGA
Il10	AGTGGAGCAGGTGAAGAGTGATTT	CTATGCAGTTGATGAAGATGTC
Il12	TCTAGATGCTGGCCAGTACACC	ATTTGGTGCTTCACACTTCAGG
Il13	GTGCCAAGATCTGTGTCTCTCC	TTACAGAGGCCATGCAATATCC
Il17	TCTGTGTCTCTGATGCTGTTGC	ACATTCTGGAGGAAGTCCTTGG
Il1T	AACCCATGATCTGGAAGAGACC	TGGTGCTGAGATAGTGTTTGTCC
Il1I	CAGGCAGTATCACTCATTGTGG	GTGCAGTTGTCTAATGGGAACG
Il6	ACAAAGCCAGAGTCCTTCAGAGA	CTGTTAGGAGAGCATTGGAAATTG
Mmp2	ACAAGTGGTCCGCGTAAAGT	GTAAACAAGGCTTCATGGGGG
Mmp3	CTCCACAGACTTGTCCCGTT	GCGCCAAAAGTGCCTGTCTT
Mmp9	ACAAGAAGTGGGGTTTCTGTCC	ATACTGGATGCCGTCTATGTCG
Mmp10	TTCAATCCCTGTATGGAGCCG	TCAGGCTCGGGATTCCAATG
Nos2	CCCTCCAGTGTCTGGGAGCA	TGCTTGTCACCACCAGCAGT
S100A8	GTCCTCAGTTTGTGCAGAATATAAA	GCCAGAAGCTCTGCTACTCC
S100A9	CTCTTTAGCCTTGAAGAGCAAG	TTCTTGCTCAGGGTGTCAGG
TgfX	AATACAGGGCTTTCGATTCAGC	TAGTTGGTATCCAGGGCTCTCC
Tnf	GTAGCCCACGTCGTAGCAAA	ACAAGGTACAACCCATCGGC
Ym1	TCTGGTGAAGGAAATGCGTAAA	GCAGCCTTGGAATGTCTTTCTC
Ym2	TGCCCCGTTCAGTTCCAT	TTGACGAAGGAATCTGATAACTGACT

METHODS

Cell lines

Cell lines were obtained from ATCC and maintained in Dulbecco's Modified Eagle's medium (DMEM) media supplemented with 10% foetal bovine serum (FCS) and 1% penicillin/streptomycin). All cell lines were negative for mycoplasma (Lonza MycoalertTM Test kit).

Animals

Animal procedures were in accordance with UK Animal law (Scientific Procedures Act 1986), including local ethics approval. $FGF2^{LMW-/-}$ mice were purchased from the Jackson Laboratory, and were bred in-house on a C57Bl/6J background ($FGF2^{tm2Doe}$/J; Stock no: 010698; Jackson Laboratory, USA). For mouse experiments, C57Bl/6J, SCID and Athymic nude mice were purchased from Charles River Laboratories. Mice were aged between 6-8 weeks for all experiments. To generate a model murine of liver metastasis, $2.0x10^5$ MC38 or KPC tumour cells were injected intrasplenically into C57Bl/6J or $FGF2^{LMW-/-}$ mice. Following injection, the spleen was removed and vessels cauterised to prevent internal bleeding. For subcutaneous tumours, murine cancer cells (MC38, KPC and LLC) were injected into the flank of C57Bl/6J ($2.0x10^5$) or $FGF2^{LMW-/-}$ mice ($1.0x10^4$). Human cancer cells (SW1222 and HT29) were injected into the flank of Athymic nude mice ($5x10^5$). To deplete T cells, mice were administered 300 µg Anti-CD3 by intra-peritoneal (I.P) injection twice weekly for 2 weeks with the initial dose one day prior to tumour cell injection (Cat No. BE0002, BioXcell, USA). For radiotherapy experiments, our irradiation regimen began once tumours reached $100mm^3$, at which point mice received 2Gy/day for 5 days, followed by 2 days of no irradiation, and then 4 additional days of 2Gy (9x2Gy). For FGF2 inhibition, GAL-F2 of anti-FGF2 mAb antibody (100 µg/dose, Galaxy Biotech, USA) was administered by I.P injection every second day for 2 weeks once tumours reached $100mm^3$. For all mouse experiments, mice were sacrificed once tumours reached $500mm^3$ or within one year of tumour induction. Tumours were measured daily in three dimensions using digital callipers, and volume was calculated using the formula 0.5 × Length × Width × Height.

Bone-marrow derived macrophage (BMDM) cultures

Femurs and tibias were harvested from mice, sterilised in 100% ethanol then placed in ice cold PBS. Bone marrow was extracted by flushing bones with 5 mL ice cold PBS, then washed twice with ice cold PBS. Following washes, bone marrow was incubated in red cell lysis buffer (RCLB) for 3 minutes to remove red blood cells, then passed through a 70 µm Nylon cell strainer and washed with ice cold PBS. Bone marrow cells ($3x10^6$) were plated

onto a 90 mm bacteriological plate in DMEM supplemented with 10% FCS and 1% penicillin/streptomycin, and 20% L929 cell supernatant to differentiate bone marrow into BMDM. Cultures were grown for 5 days prior to experimental use. BMDM were treated with 100 ng/mL LPS (Cat No. L4130, Sigma) or 20 ng/mL IL-4 (Cat No. 214-14, PetroTech, USA) to alter polarisation. For co-cultures, $5x10^5$ cancer cells were plated in cell culture inserts (Cat No. PICM03050, Millicell, USA) 1 day prior to use. 3 inserts were placed in each plate. FGF2 (100 ng/ml of recombinant human FGF2; Biolegend,USA Cat.710304) was added for 1h in serum-free medium to the BMDM 5 days after bone marrow cells were seeded and incubated for an additional 24 h in 1% serum before harvest. In order to evaluate the effect of MC38 cell conditioned medium, medium for BMDM was changed from bone marrow differentiation medium to MC38 conditioned medium, added as a mixture of 50 % MC38 conditioned medium and 50 % bone marrow differentiation medium 5 days after the bone marrow cells were seeded. To generate conditioned medium, MC38($1x10^6$ cells) were grown in a T175 flask over approximately 4-5 days until they reached 70-80% of confluence. Then the medium was collected, centrifuged at 1200 rpm for 5 min to collect the supernatant. After incubation of the BMDM in the MC38 conditioned mediums, recombinant human FGF2 was added to the BMDM in serum free medium for 1 h and incubated for an additional 24h under serum-starvations. Cells were harvested and lysed for further analysis.

Histochemistry

Liver lobes and subcutaneous tumours were embedded in optical cutting temperature (OCT) media and 10 µm sections were sectioned and stored at -30˚C. Prior to staining, sections were dried overnight at 37˚C. For H&E staining, slides were hydrated in graded ethanol solutions then placed in Heamatoxylin Harris (Cat No. 3519455, VMR Chemicals, USA) for 2 minutes, rinsed in tap water and differentiated in 1% hydrochloride in 70% ethanol for 30 seconds. Following differentiation, samples were washed in tap water then incubated in Eosin solution (Cat No. HT110132, Sigma, USA,) for 2 minutes before being dehydrated in graded ethanol. Slides were incubated in xylene before being mounted with Vecta Mount (Cat No. H-5000, VectorLabs, USA). For immunofluorescence, slides were fixed in 4% paraformaldehyde or 100% acetone or 100% methanol and permeabilized with 0.1% Triton. Slides were blocked with 20% goat serum for 1 h at room temperature (RT) and primary antibodies were incubated for 2 h at room temperature. Slides were washed and secondary antibodies were added for 1 hour at RT in the dark. Slides were then stained with Hoechst (1 in 250, 5mg/mL, Cat No. 33342, ThermoFisher, USA) for 10 minutes and mounted with ProLong Diamond Antifade Mountant (Cat No. P36961, ThermoFisher). Immunofluorescence images were captured with a Leica DM6000 confocal microscope or

Zeiss 880 two-photon microscope and analyzed with ZEN (Zeiss, Germany) and ImageJ. Antibodies were used at the following concentrations; Goat Anti-mouse CD3 (M-2) (Cat No. SC-1127, Santa Cruz Biotechnology, USA); Rat Anti-Mouse CD4 (GK1.5) (Cat No. SC13573, Santa Cruz Biotechnology); Rat Anti-Mouse CD8 (2.43) (Cat No. SC18860, Santa Cruz Biotechnology); Rat Anti-Mouse F4/80 (A3-1) (Cat No. ab6640, Abcam); Mouse Anti-Mouse FGF2 (bFM-2) (Cat No. 05-118, Merck, USA).

Flow cytometry and cell sorting

Subcutaneous tumours were harvested, washed with PBS and incubated in Hanks Balance Salt Solution (HBSS) media supplemented with Collagenase II and DNase I for 30 minutes at 37°C. Following incubation, samples were passed through a 70 µm Nylon cell strainer, washed twice with PBS before incubation RCLB for 3 minutes to remove red cells. After the RCLB, samples were washed twice with PBS and passed through a 70 µm Nylon cell strainer. Samples were then suspended in ice cold FACs buffer (3% FCS in PBS) for surface marker staining. For intracellular staining, samples were fixed/permeabilized using the eBioscience Foxp3/Transcription Factor Staining Buffer Set (Cat No. 00-5523-00, eBioscience, USA). To analyze FGF2 expression immune cells, and cytokine expression in T cells, samples were incubated at 37°C with GolgiSTOP™ (1 in 1000; Cat No. 554724, BD Bioscience, USA) and GolgiPlug™ (1 in 1000; Cat No. 555029, BD Bioscience) for 3 hours to block secretion prior to staining. For flow cytometry, samples were ran through a LSRII Flow Cytometer (BD Bioscience) and analysis was performed using FlowJo V10 software. For cell sorting, samples were ran through an Aria II Cell Sorter (BD Bioscience). Refer to **Supplementary Table 1** for flow cytometry antibody information.

RNA extraction and Quantitative RT-PCR (qRT-PCR) analysis

For tissue samples, RNA was harvested using TRIzol reagent in accordance with the manufacturer's guidelines (Cat No. 15596026, Life Technologies, USA). For cell sorted samples, RNA was harvested using RNeasy® Mini Kit (Cat No. 74104, Qiagen, USA). 50 ng-1 µg RNA was reverse transcribed using Moloney murine leukemia virus reverse transcriptase (M1701, Promega, USA) primed with oligo (dT) (Cat No. C1101, Promega). Quantitative PCR (QPCR) primers were designed using PRIMER EXPRESS (Applied Biosystems, USA). SYBR green chemistry was used with rL32 or beta actin as the internal reference gene. The conditions were 95°C for 10 minutes, 40 cycles of 95°C for 30 seconds and 60°C for 1 minutes (QuantStudio5, ThermoFisher, USA). Relative fold differences were determined using the $\Delta\Delta$Ct method. Refer to **Supplementary Table 2** for flow cytometry antibody information.

GSEA analysis

Gene Set Enrichment Analysis (GSEA) (reference PMID 16199517) was performed using the Broad Institute desktop application (http://software.broadinstitute.org/gsea/downloads.jsp) on RNA-Seq gene expression data from the TCGA colorectal cancer dataset. Phenotype for the FGF pathway was defined on the basis of FGF2, FGFR1, FGFR2 and MAPK3 (ERK1) expression. Z-scores were generated for the expression of each gene across the whole patient cohort and a z-score ≥1.5 was used to define FGF pathway expression. Genesets indicative of specific immune cell infiltrate were obtained from a publication identifying specific 'immunomes' within cancer (reference PMID 24138885) and enrichment for these genesets analysed in the FGF phenotype dataset.

Clonogenic Assay

Cells were seeded in 6-well plates with a density of 2 to 10 $\times 10^2$ cells/well and maintained at 37°C with 5% CO_2. Twenty-four hours later, GAL-F2 of anti-FGF2 mAb antibody (100 μg/dose, Galaxy Biotech, USA), recombinant FGF2 (applied at 100ng/μg protein, Biolegend, USA; Cat. 710304) or BGJ398 (pan FGFRs inhibitor, Cat No. S2183, Selleckchem, USA) was added to cells at indicated concentration and treated with gamma-rays. Gamma-ray treatment was delivered using a ^{137}Cs-laboratory irradiator (IBL 637; CIS bio International, Saclay, France), which produces gamma-rays (0.662 MeV) at a dose rate of 0.81 Gy/min. Viability and plating efficiency were also evaluated for tumour cells.

Western Blotting

Cells were incubated in protease inhibitor (Cat No.5871S, Cell Signalling, USA) diluted in radioimmunoprecipitation assay buffer (Cat No. R0278, Sigma-Aldrich, USA)) for 30 minutes at 4°C before centrifugation at 15,000rpm for 20 minutes. Protein concentration was confirmed by bicinchoninic acid assay kit (Cat No. 23225, ThermoFisher Scientific, UK). Samples were run on 4%-12% Bis-Tris gels for 90 minutes at 120 V. Following transfer, nitrocellulose membranes were blocked in 5% milk before incubation overnight at 4°C in a 2% milk solution containing antibodies. Antibodies were *p*-AKT (Thr368), *p*-AKT (Ser473), AKT, p-ERK, ERK (Cell Signaling Technology, USA) and FGF2 (Cat No. 05-118, Merck, USA). After washing, membranes were incubated horseradish-peroxidase–conjugated secondary antibodies before being developed in enhanced chemiluminescence and visualized using the Odyssey Imaging System (Licor, USA).

Vascular analysis

Tumour sections mounted on slides were blocked with 20% goat serum for 1 h at room temperature (RT) and incubated with rat anti-CD31 antibody (Abcam, Cat. Ab7388, 1:100) for 2 h at room temperature. Slides were washed and anti-rat Alexa fluor 546 antibody was applied (Invitrogen, Cat. A-11081, 1:200). Whole tumour and vascular images were obtained under the tile scanning mode using a Zeiss 880 two-photon microscope (10X). The images were analysed with ImageJ. Vascular size was measured with area stained with CD31 conjugated with Alexa fluor 546. Details of mice and vessel numbers are shown in supplementary figure-5E table.

Statistical Analysis

All data is expressed as mean ± S.D and statistical analysis was performed by one-way analysis of variance (ANOVA), the appropriate parametric (student T test) statistical test or non-parametric data (Mann–Whitney) using SigmaStat (Jandel Scientific). P-values ≤ 0.05 were considered statistically significant.

Data Availability Statement

The data that support the findings of this study are available from the corresponding author upon reasonable request. Images of the gels used in figures are available in the supplementary information.

1. p-Akt (Thr308, Cell Signaling, Cat.13038)
2. P-Akt (Ser473, Cell Signaling, Cat.4060L)
3. P-ERK (Cell Siganling, Cat.4377S)
4. GAPDH (abcam, Cat.ab8245)
5. FGF2 (Merck, Cat.05-118)

Supplemental Figure 7C

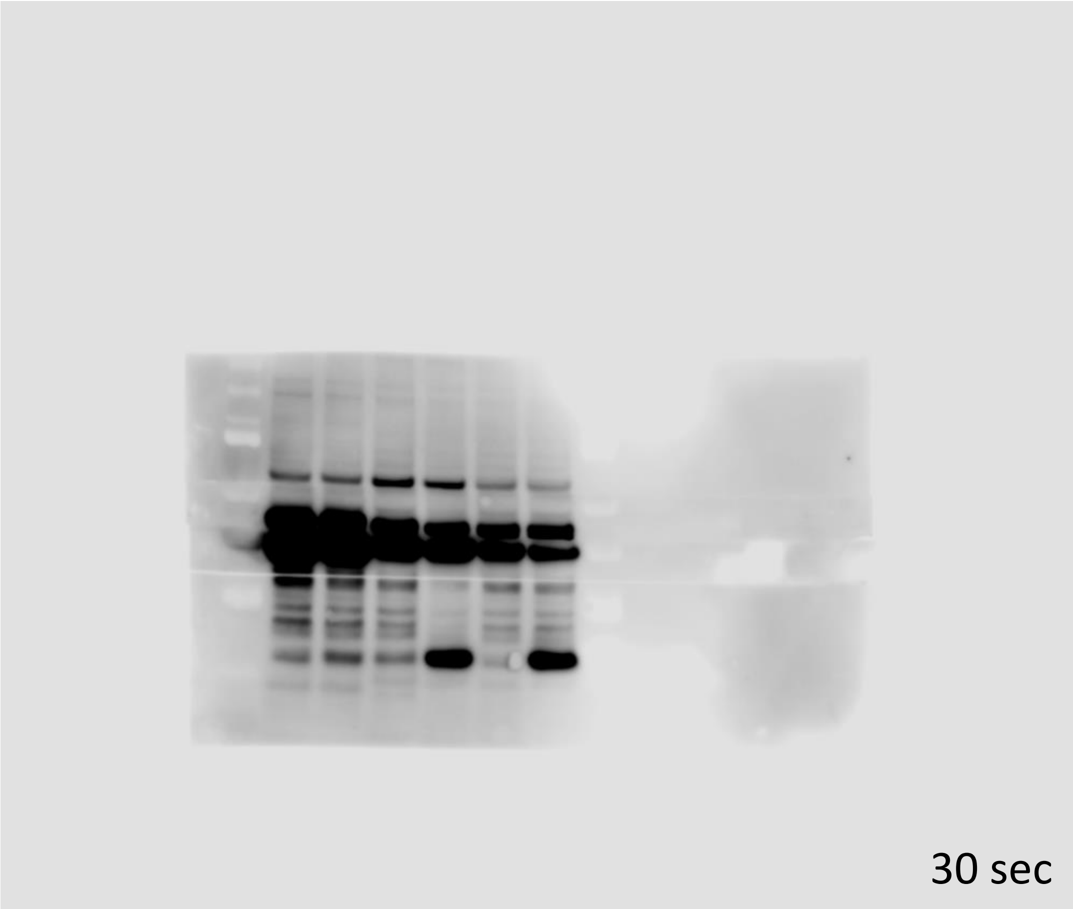

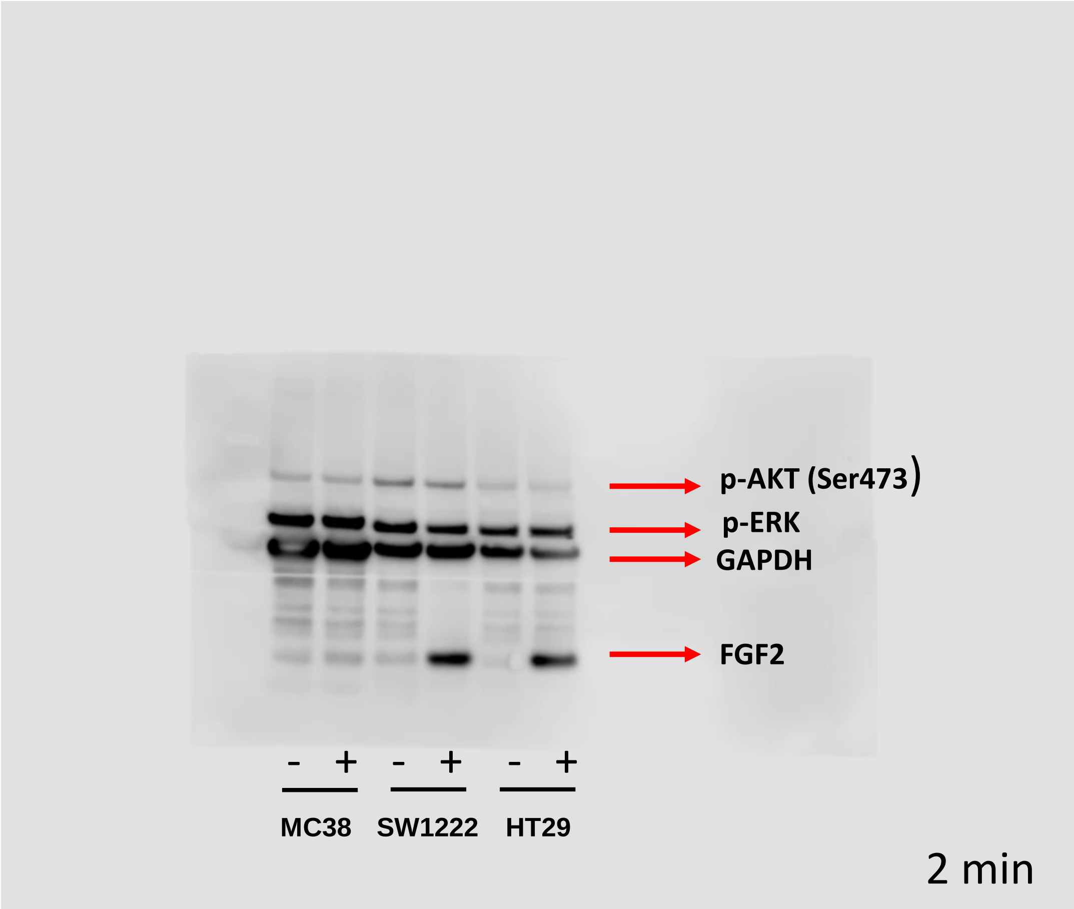

Supplemental Figure 7D-1

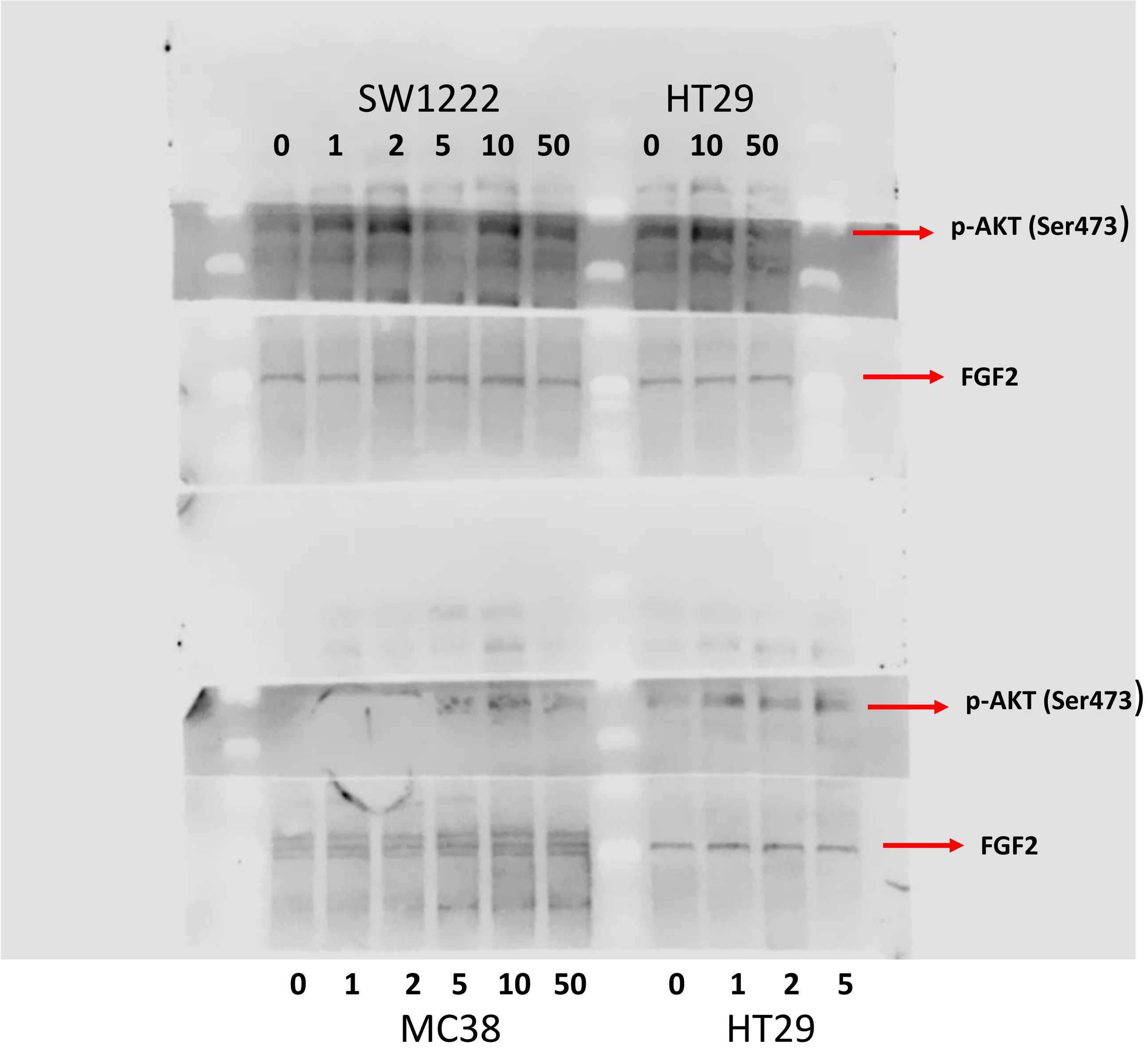

Supplemental Figure 7D-2

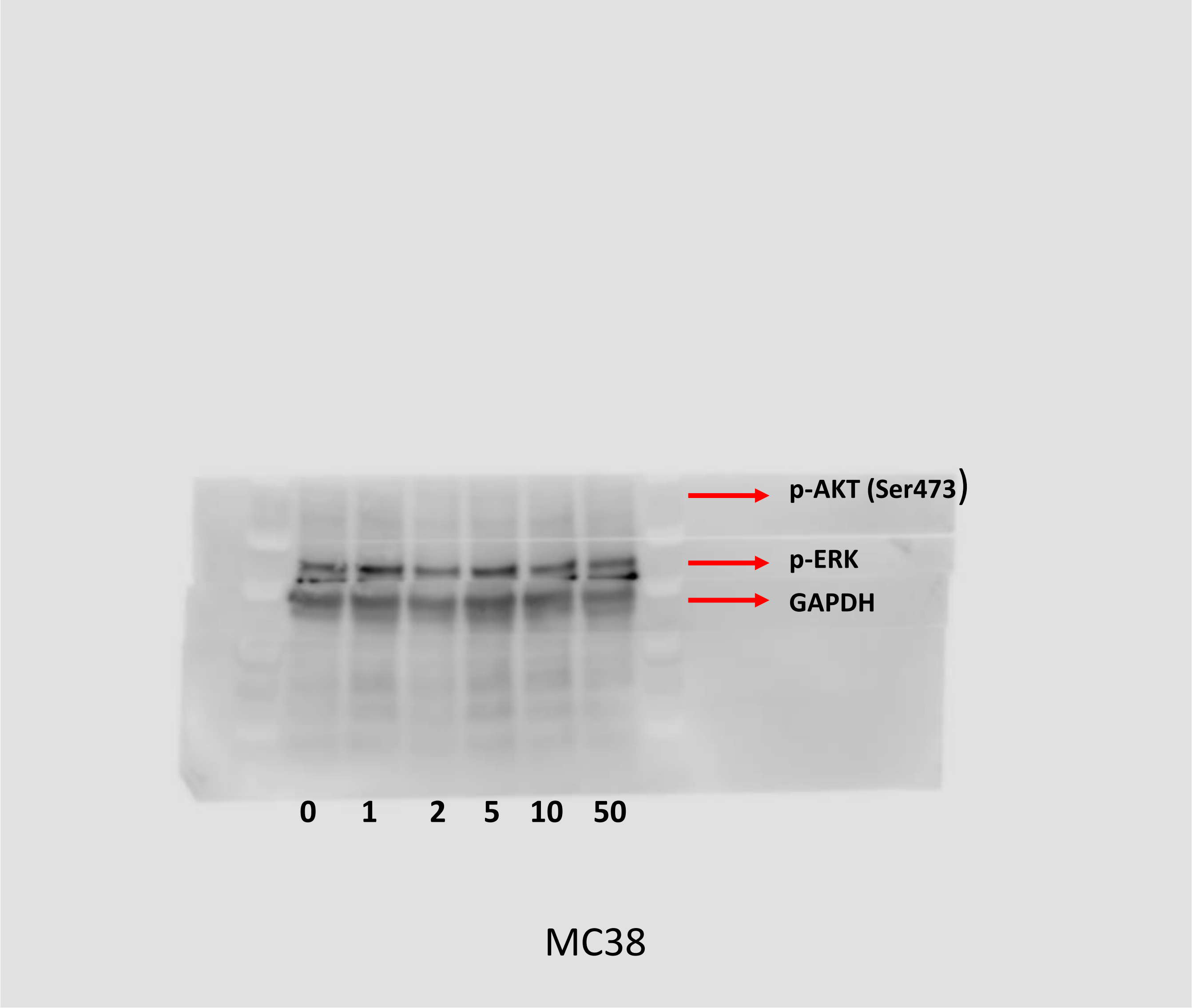

Supplement: Supplementary file 1 — Supplementary Information [file 41467_2020_17914_MOESM1_ESM.pdf]
