# Supplementary material for: FGF2 alters macrophage polarization, tumour immunity and growth and can be targeted during radiotherapy
Source: Nat Commun. 2020 Aug 13;11:4064. doi: 10.1038/s41467-020-17914-x (PMC7426415; doi:10.1038/s41467-020-17914-x)
Supplement: Supplementary file 3 — Reporting Summary [file 41467_2020_17914_MOESM3_ESM.pdf]

nature research

Corresponding author(s): Ruth J Muschel

Last updated by author(s): 21 June 2020

Reporting Summary

Nature Research wishes to improve the reproducibility of the work that we publish. This form provides structure for consistency and transparency in reporting. For further information on Nature Research policies, see our Editorial Policies and the Editorial Policy Checklist.

Statistics

For all statistical analyses, confirm that the following items are present in the figure legend, table legend, main text, or Methods section.

n/a	Confirmed	
☐	☒	The exact sample size (n) for each experimental group/condition, given as a discrete number and unit of measurement
☐	☒	A statement on whether measurements were taken from distinct samples or whether the same sample was measured repeatedly
☐	☒	The statistical test(s) used AND whether they are one- or two-sided<br>*Only common tests should be described solely by name; describe more complex techniques in the Methods section.*
☐	☒	A description of all covariates tested
☐	☒	A description of any assumptions or corrections, such as tests of normality and adjustment for multiple comparisons
☐	☒	A full description of the statistical parameters including central tendency (e.g. means) or other basic estimates (e.g. regression coefficient) AND variation (e.g. standard deviation) or associated estimates of uncertainty (e.g. confidence intervals)
☐	☒	For null hypothesis testing, the test statistic (e.g. F, t, r) with confidence intervals, effect sizes, degrees of freedom and P value noted<br>*Give P values as exact values whenever suitable.*
☒	☐	For Bayesian analysis, information on the choice of priors and Markov chain Monte Carlo settings
☒	☐	For hierarchical and complex designs, identification of the appropriate level for tests and full reporting of outcomes
☒	☐	Estimates of effect sizes (e.g. Cohen's d, Pearson's r), indicating how they were calculated

Our web collection on statistics for biologists contains articles on many of the points above.

Software and code

Policy information about availability of computer code

Data collection	No software used except for that built into the indicated equipment..
Data analysis	ZEN (Zeiss, Germany): Image analysis obtained from Zeiss 880 two-photon microscope.<br>ImageJ: Image analysis obtained from western blot and immunohistochemstry<br>IMARIS (Oxford Instrument, UK): Image analysis obtained from Zeiss 880 tow-photon microscope.<br>FlowJo V10(BD, USA): Analysis obtained from Flow cytometer<br>The Broad Institute desktop application (http://software.broadinstitue.org/gsea/downloads.jsp): GSEA analysis (referece PMID 16199517)<br>SigmaStat(Jandel Scientific): Statistical analysis

For manuscripts utilizing custom algorithms or software that are central to the research but not yet described in published literature, software must be made available to editors and reviewers. We strongly encourage code deposition in a community repository (e.g. GitHub). See the Nature Research guidelines for submitting code & software for further information.

Data

Policy information about availability of data

All manuscripts must include a data availability statement. This statement should provide the following information, where applicable:

- Accession codes, unique identifiers, or web links for publicly available datasets
- A list of figures that have associated raw data
- A description of any restrictions on data availability

The data that support the findings of this study are available from the corresponding author upon reasonable request. Images of the gels used in figures are available in the supplementary information.

Field-specific reporting

Please select the one below that is the best fit for your research. If you are not sure, read the appropriate sections before making your selection.

[x] Life sciences   [ ] Behavioural & social sciences   [ ] Ecological, evolutionary & environmental sciences

For a reference copy of the document with all sections, see nature.com/documents/nr-reporting-summary-flat.pdf

Life sciences study design

All studies must disclose on these points even when the disclosure is negative.

Sample size	Is explained in the text. Otherwise at least 3 experimental replicates and n=5 for each group in murine experiments. Power calculations were used for the initial design of murine experiments, but may have been modified as more information was gained.
Data exclusions	No data was excluded from analysis.
Replication	All attempts at replication were successful.
Randomization	Mice were recruited into the study by rotation as their tumours reached the indicated sizes. Mice were culled when their tumours reached the indicated sizes.
Blinding	Investigators were blinded to group allocation during data collection and analysis.

Reporting for specific materials, systems and methods

We require information from authors about some types of materials, experimental systems and methods used in many studies. Here, indicate whether each material, system or method listed is relevant to your study. If you are not sure if a list item applies to your research, read the appropriate section before selecting a response.

Materials & experimental systems

n/a	Involved in the study	
[ ]	[x]	Antibodies
[ ]	[x]	Eukaryotic cell lines
[x]	[ ]	Palaeontology and archaeology
[ ]	[x]	Animals and other organisms
[x]	[ ]	Human research participants
[x]	[ ]	Clinical data
[x]	[ ]	Dual use research of concern

Methods

n/a	Involved in the study	
[x]	[ ]	ChIP-seq
[ ]	[x]	Flow cytometry
[x]	[ ]	MRI-based neuroimaging

Antibodies

Antibodies used

1. Flow Cytometry

LIVE/DEAD™ Fixable Violet Dead Cell Stain Kit, for 405 nm excitation (Life Technologies, Cat.L34955)
Alexa Fluor® 700 anti-mouse CD45 Antibody (BioLegend, Cat.103127)
CD8a Monoclonal Antibody (53-6.7), PE-Cyanine7 (eBiosource, Cat.25-0081-82)
CD11c Monoclonal Antibody (N418), PE (eBiosource, Cat.12-0114-82)
Ly-6G (Gr-1) Monoclonal Antibody (RB6-8C5), PerCP-Cyanine5.5 (eBiosource, Cat.45-5931-80)
CD3e Monoclonal Antibody (145-2C11), APC (eBiosource, Cat.17-0031-82)
Brilliant Violet 510™ anti-mouse/human CD11b Antibody (BioLegend, Cat.101245)
Brilliant Violet 605™ anti-mouse CD4 Antibody (BioLegend, Cat.100547)
APC/Cy7 anti-mouse F4/80 Antibody (BioLegend, Cat.123117)
bFGF Polyclonal Antibody, FITC Conjugated (Bioss, Cat.bs-0217R-FITC)
FGF R1 Antibody (M19B2) [Alexa Fluor® 488] (Novus, Cat.NB600-1287AF488)
FGFR2 Polyclonal Antibody (conguated to alexa 488) (ThermoFisher, Cat.PA5-14651)
iNOS Monoclonal Antibody (CXNFT), PE (eBiosource, Cat.12-5920-82)
PerCP/Cy5.5 anti-mouse CD206 (MMR) Antibody (BioLegend, Cat.141716)

2. Immunofluorescence histochemistry

Goat anti-mouse CD3(M-2) antibody (Santa Cruz Biotechnology, Cat.SC1127)
Rat anti-mouse CD4(GK1.5) antibody (Santa Cruz Biotechnology, Cat.SC13573)

Rat anti-mouse CD8(2.43) antibody (Santa Cruz Biotechnology, Cat.SC18860)
Rat anti-mouse F4/80(A3-1) antibody (Abcam, Cat.ab6640)
Mouse anti-mouse FGF2(bFM-2) antibody (Merck, Cat.05-118)
Rat anti-mouse CD31 antibody (Abcam, Cat.ab7388)

3. In vivo experiment

Anti CD3 antibody (BioXcell, Cat.BE0002)
Anti FGF2 antibody (Galaxy Biotech)
Anti CD31 antibody conjugated with Phycoerythrin (Biolegend, Cat.102408)

4. Western Blot

Rabbit polyclonal anti-GAPDH antibody (Abcam, Cat. ab9485)
Rabbit monoclonal anti-p-AKT(Thr308) antibody (Cellsignaling Technology, Cat.13038)
Rabbit monoclonal anti-p-AKT(Ser473) antibody (Cellsignaling Technology, Cat.4060)
Rabbit monoclonal anti-p-MAPK (ERK1/2) antibody (Cellsignaling Technology, Cat.4377)
Mouse anti-mouse FGF2(bFM-2) antibody (Merck, Cat.05-118)

Validation: Validation of antibodies used in all experiments was indicated at manufacturer's websites.

Eukaryotic cell lines

Policy information about cell lines

Cell line source(s)	American Type Culture Collection (ATCC)
Authentication	Yes
Mycoplasma contamination	All cell lines were negative for mycoplasma (Lonza MycoalertTM test kit) within 2 months of use. We have not had any detected mycoplasma contamination in any cell line during the time when these studies were conducted.
Commonly misidentified lines (See ICLAC register)	N/A

Animals and other organisms

Policy information about studies involving animals; ARRIVE guidelines recommended for reporting animal research

Laboratory animals	C57Bl/6J: female, 6-8 weeks, Charles River<br>SCID: female, 6-8 weeks, Charles River<br>Athymic nude mice: female, 6-8 weeks, Charles River<br>FGF LMW-/- mice (FGF2 tm2Doe/J): female, 6-8 weeks, Stock no:010698, Jackson Laboratory)
Wild animals	N/A
Field-collected samples	N/A
Ethics oversight	UK Animal law (Scientific Procedure Act 1986)

Note that full information on the approval of the study protocol must also be provided in the manuscript.

Flow Cytometry

Plots

Confirm that:

- [x] The axis labels state the marker and fluorochrome used (e.g. CD4-FITC).
- [x] The axis scales are clearly visible. Include numbers along axes only for bottom left plot of group (a 'group' is an analysis of identical markers).
- [x] All plots are contour plots with outliers or pseudocolor plots.
- [x] A numerical value for number of cells or percentage (with statistics) is provided.

Methodology

Sample preparation: Subcutaneous tumours were harvested, washed with PBS and incubated in Hanks Balance Salt Solution (HBSS) media supplemented with Collagenase II and DNase I for 30 minutes at 37°C. Following incubation, samples were passed through a

	70 µm Nylon cell strainer, washed twice with PBS before incubation RCLB for 3 minutes to remove red cells. After the RCLB, samples were washed twice with PBS and passed through a 70 µm Nylon cell strainer. Samples were then suspended in ice cold FACs buffer (3% FCS in PBS) for surface marker staining. For intracellular staining, samples were fixed/permeabilized using the eBioscience Foxp3/Transcription Factor Staining Buffer Set (Cat No. 00-5523-00, eBioscience, USA). To analyze FGF2 expression immune cells, and cytokine expression in T cells, samples were incubated at 37°C with GolgiSTOP™ (1 in 1000; Cat No. 554724, BD Bioscience, USA) and GolgiPlug™ (1 in 1000; Cat No. 555029, BD Bioscience) for 3 hours to block secretion prior to staining.
Instrument	LSRII Flow Cytometer (BD Bioscience)
Software	FlowJo V10(BD, USA)
Cell population abundance	Is described in the text and the reference provided in the Supplementary text from our previous work with this same method. Jones et al.<br>doi: 10.15252/emmm.201809342
Gating strategy	Is based upon a reference by our lab provided in the Supplementary text. Jones et al. The figure is in that paper. doi: 10.15252/emmm.201809342 This paper shows the gating strategy.

☐ Tick this box to confirm that a figure exemplifying the gating strategy is provided in the Supplementary Information.
